# Early white matter changes in CADASIL: evidence of segmental intramyelinic oedema in a pre-clinical mouse model

**DOI:** 10.1186/2051-5960-2-49

**Published:** 2014-04-30

**Authors:** Emmanuel Cognat, Sabine Cleophax, Valérie Domenga-Denier, Anne Joutel

**Affiliations:** INSERM, U1161 and Univ Paris Diderot, Sorbonne Paris Cité, UMRS 1161, F-75010 Paris, France

**Keywords:** Small vessel disease, White matter, Intramyelinic oedema, CADASIL, Mouse model

## Abstract

**Introduction:**

Small vessel disease (SVD) of the brain is a leading cause of age- and hypertension-related cognitive decline and disability. Cerebral white matter changes are a consistent manifestation of SVD on neuroimaging, progressing silently for many years before becoming clinically evident. The pathogenesis of these changes remains poorly understood, despite their importance. In particular, their pathological correlate at early stages remains largely undefined. Cerebral Autosomal Dominant Arteriopathy with Subcortical Infarcts and Leukoencephalopathy (CADASIL), caused by dominant mutations of the NOTCH3 receptor, is regarded as a paradigm for the most common form of sporadic SVD. In this study, we used immunohistochemistry, confocal microscopy and electron microscopy, together with qualitative and quantitative analyses to assess oligodendroglial, axon and myelin damage in TgPAC-Notch3^R169C^ mice, a model of preclinical CADASIL.

**Results:**

The principal cerebral white matter changes in TgPAC-Notch3^R169C^ mice are microvacuoles (≤1 μm diameter) in the myelin sheaths associated with focal myelin degradation and occurring in the absence of oligodendrocyte loss. Half the damaged myelin sheaths still contain an apparently intact axon. Clearance of myelin debris appears inefficient, as demonstrated by the significant but mild microglial reaction, with occasional myelin debris either contacted or internalized by microglial cells.

**Conclusion:**

Our findings suggest that segmental intramyelinic oedema is an early, conspicuous white matter change in CADASIL. Brain white matter intramyelinic oedema is consistently found in patients and mouse models with compromised ion and water homeostasis. These data provide a starting point for novel mechanistic studies to investigate the pathogenesis of SVD-related white matter changes.

**Electronic supplementary material:**

The online version of this article (doi:10.1186/2051-5960-2-49) contains supplementary material, which is available to authorized users.

## Introduction

Cerebral small vessel disease (SVD) is increasingly recognized as a major health problem in developed countries, accounting for 25 to 30% of ischemic stroke [1] and as a leading cause of age-related cognitive decline and disability [2]. The majority of SVD appears to result from a complex mix of genetic and cardiovascular risk factors, the most important of which are age and hypertension [3, 4].

The main manifestations of SVD on standard magnetic resonance imaging (MRI) are lacunes and white matter (WM) hyperintensities [5]. The underlying microstructural changes responsible for WM hyperintensities are unclear, and it has been suggested that these features may simply reflect an increase in water content [6]. Novel MRI techniques, such as diffusion tensor imaging (DTI), which measures the magnitude and direction of water diffusion, and magnetization transfer imaging (MTI), which measures the efficiency of magnetization exchange between the relatively free water protons within tissue and protons bound to macromolecules, are highly sensitive to changes in white matter tract integrity [7, 8]. Changes reflecting axonal loss and demyelination have consistently been observed in subjects with SVD. These changes are detected not only in areas of WM hyperintensities but also in white matter of normal appearance, suggesting that the WM hyperintensities observed on conventional MRI may be no more than the tip of the iceberg [6]. WM lesions are highly prevalent in patients with SVD, and in populations at risk of SVD, including the elderly, in particular. This suggests that WM lesions probably progress silently for many years before becoming clinically evident [9].

Little is known about the pathophysiological events leading to WM lesions in SVD. The two prevailing hypotheses attribute WM abnormalities to chronic hypoperfusion [3], or to an increase in blood–brain-barrier permeability, causing fluid to leak into the surrounding brain tissue [4]. It has also been suggested that WM changes may arise from Wallerian degeneration due to cortical neuron loss [10]. There are several reasons for the current lack of knowledge. Firstly, SVD is highly heterogeneous and the mechanisms underlying WM lesions are potentially diverse [3]. Secondly, the pathological correlate of WM abnormalities, particularly at early stages, remain largely undefined. WM lesions have been studied on autopsy, in patients with end-stage SVD [4, 9]. At this stage, severe white matter disorganization is the rule, with marked losses of myelin and axons, accompanied by severe vessel wall and perivascular space abnormalities [11].

Monogenic forms of SVD, largely indistinguishable from sporadic SVD, have been characterized in recent years [12]. Cerebral Autosomal Dominant Arteriopathy with Subcortical Infarcts and Leukoencephalopathy (CADASIL), caused by dominant mutations in the NOTCH3 receptor, is regarded as a paradigm for the most common form of sporadic SVD [13–15]. All *NOTCH3* mutation carriers display WM abnormalities, which constitute early signs of the disease detectable well before the occurrence of stroke, cognitive impairment and disability [16]. In the current study, we investigated a mouse model of preclinical CADASIL (TgPAC-Notch3^R169C^), in which cerebral WM pathology develops with aging [17, 18]. Through immunohistochemistry, confocal microscopy and electron microscopy, combined with qualitative and quantitative evaluations, we carried out, to the best of our knowledge, the first microstructural characterization of changes to brain WM in the initial stages of a pure, well defined form of SVD.

## Material and methods

### Mice

TgPAC-Notch3^R169C^ (FVB/N) and TgPAC-Notch3^WT^ (FVB/N) mice have been described elsewhere [17]. These mice and their wild-type control littermates were bred at the animal facility on the Villemin site of Paris Diderot University (Paris, France). The TgPAC-Notch3^WT^ mice and their non-transgenic littermates did not differ for any of the parameters assessed, and we therefore pooled the data for these two groups, which we used as a single control group. Mice were housed under a normal light/dark cycle (12 h) with standard rodent chow and tap water supplied *ad libitum*. All the experiments described here were conducted in full accordance with the guidelines of our local institutional animal care and use committee (Lariboisière-Villemin), and every effort was made to minimize the number of animals used.

### Electron microscopy

Mice were deeply anesthetized with sodium pentobarbital (80 mg/kg) and transcardially perfused with 50 ml of 0.1 M sodium phosphate buffer (PB) followed by 200 ml of 2% glutaraldehyde/2% paraformaldehyde in PB. The skull was opened and incubated for one hour in 2% glutaraldehyde/2% paraformaldehyde in PB at 4°C, for fixation of the brain tissues. The brain was removed, cut into 1 mm-thick paramedian brain slices (median, −1 mm, +1 mm), and 1×1×1-mm blocks from the corpus callosum, internal capsule and fimbria regions were dissected under a microscope. The blocks of tissue were incubated overnight in 2% glutaraldehyde/2% paraformaldehyde in PB at 4°C and then post-fixed by incubation in 2% osmium tetroxide in PB. Then samples were dehydrated by passage through a graded series of ethanol concentrations and were then embedded in epoxy resin. Semi-thin sections were cut with an ultramicrotome (Leica EM EC7), stained with 1% toluidine blue and screened by light microscopy to select areas in which the myelin fibers were perpendicular to the cutting plane. Ultrathin sections of regions of interest were cut, mounted on copper grids, contrast stained with uranyl acetate and lead citrate and examined by transmission electron microscopy (Philips CM100).

### Immunohistochemistry

Mice were deeply anesthetized with sodium pentobarbital (80 mg/kg) and transcardially perfused with 50 ml of PB followed by 50 ml of 4% paraformaldehyde in PB. The brain was removed, cut along the midline and postfixed by incubation overnight in 4% paraformaldehyde at 4°C. Half the brain was dehydrated, embedded in paraffin, and cut into 7 μm-thick sagittal sections on a rotary microtome (Leica RM2255). These sections were placed on silane-treated glass slides. The other half of the brain was cryoprotected by incubation in 30% sucrose solution until precipitation occurred at 4°C, transferred to a plastic cryomold filled with optimal cutting temperature compound (Tissue-tek) and immersed in a pre-chilled isopentane bath cooled with liquid nitrogen. It was then (immediately before use) cut into 16 μm-thick sagittal slices on a cryostat (Leica CM 1850), and the slices were collected in PBS in 12-well plates.

Immunodetection for myelin basic protein, Iba-1, CD68, Alzheimer precursor protein A4, phosphorylated light neurofilament, non-phosphorylated heavy neurofilament and cleaved caspase 3 was performed on free-floating cryosections. Sections were washed and blocked by incubation with 5% bovine serum albumin (BSA) in PBS supplemented with 0.4% Triton X-100 for 1 h at room temperature. They were then incubated in 0.5% BSA and 0.4% Triton X-100 at 4°C overnight, with one or two of the following primary antibodies: mouse monoclonal anti-myelin basic protein (1:2000, SMI94, Covance), rabbit polyclonal anti-degraded myelin basic protein (1:500, Millipore), rabbit polyclonal anti-Iba-1 (1:2000, Wako), rat monoclonal anti-CD68 (1:250, FA-11, AbD Serotec), rabbit monoclonal anti-phosphorylated neurofilament light (1:100, C28E10, Cell Signaling), mouse monoclonal anti-non-phosphorylated neurofilament heavy (1:50000, SMI32, Covance), mouse monoclonal anti-Alzheimer precursor protein A4 (1:1000, 22C11, Millipore), rabbit monoclonal anti-cleaved caspase 3 (1:250, Asp175/5A1E, Cell Signaling). Olig2 immunodetection was performed on paraffin-embedded sections. Samples were deparaffinized, rehydrated in graded series of ethanol concentrations and boiled in 10 mM citrate buffer (pH 6.0) for 10 minutes to unmask the antigens. The slides were allowed to cool to room temperature and were washed in PBS. Sections were blocked by incubation with 5% fetal bovine serum in PBS supplemented with 0.2% Triton X-100 for 1 h at room temperature. They were then incubated with a rabbit polyclonal anti-Olig2 antibody (1:200, Millipore) in 0.5% fetal bovine serum, overnight at 4°C.

The cryosections and paraffin-embedded sections were then incubated with AlexaFluor-conjugated secondary antibodies (1:500, Life technologies) for 2 h at room temperature. Sections were washed, counterstained with DAPI (1:10,000, Sigma-Aldrich) in PBS for 5 minutes at room temperature, mounted in a drop of Dako fluorescence mounting medium and subjected to epifluorescence imaging (Nikon eclipse 80i) or laser scanning confocal microscopy (Olympus BX61). The same imaging parameters were used for all images acquired from all the groups compared. A detailed description of all the primary and secondary antibodies used is provided in Additional file 1: (Table S1).

### Quantitative analysis

All quantitative image analyses were performed in blind conditions.

#### Lesions on electron micrographs

Lesions were first counted in the corpus callosum when the mice were 20 months old (*n* = 4 TgPAC-Notch3^R169C^ mice and 4 control mice). Images were acquired randomly, with a magnification of x3,400 (6 fields per section, 1–2 sections per mouse). A 400 μm^2^ square was randomly drawn on each image and the lesions located within the square (excluding 2 borders) were counted manually. Results are expressed as the number of lesions per 1000 μm^2^. The lesions present in TgPAC-Notch3^R169C^ mice were characterized further. A mean of 200 lesions per section (from 7–12 fields, 1–2 sections per mouse, *n* = 4 mice) were manually and systematically identified with NIH ImageJ software and assessed to determine the lesion type, its subcellular location, its position relative to the myelin sheath, and the presence or absence of an axon. Degenerating axons were distinguished on the basis of an accumulation of mitochondria and lysosomes in the axoplasm, vacuolar bodies, a disrupted plasma membrane, or a dark axoplasm. The other axons were considered to be “apparently intact”. In vacuolated lesions, area and equivalent diameter were measured. The results are expressed as a percentage of the total lesion count.

#### g-ratios

The g-ratio (diameter of the axon/total outer myelin sheath diameter) was determined on electron micrographs of the corpus callosum of 20-month old mice (*n* = 3 TgPAC-Notch3^R169C^ mice and 3 control mice). Images were acquired randomly at a magnification x17,500. The g-ratio was calculated for at least 120 randomly selected apparently intact myelin sheaths (i.e., non-vacuolated) per genotype, with the GRatio for ImageJ plugin (Ingo Bormuth, http://gratio.efil.de/). We then plotted g-ratio against axon diameter.

#### Myelin debris

The amount of myelin debris was determined at the ages of 12 months (*n* = 4 TgPAC-Notch3^R169C^ mice and 4 control mice) and 20 months (*n* = 5 TgPAC-Notch3^R169C^ mice and 8 control mice). Epifluorescence images were obtained for sections (1.5 ± 0.4 mm lateral to the midline) labeled with SMI94, with a 20x objective, from both the anterior (2 fields) and posterior (1 field) parts of the corpus callosum (3 fields per section, 4–10 sections per mouse), using a CCD camera and identical parameters. Myelin debris were counted with a custom-made three-step NIH ImageJ macro: manual delineation of the corpus callosum, automatic detection of the foci with the highest intensity relative to the regional background (local maxima) and automated counting of hyperintense foci and measurement of the area of the corpus callosum. The results are expressed as the number of SMI94 hyperintense foci over the area of the corpus callosum.

#### Presence of axons within damaged myelin fibers

We checked altered myelin sheaths for the presence of the axon in 20-month-old TgPAC-Notch3^R169C^ mice (*n* = 5). Confocal images of the fimbria were generated with a x60 oil-immersion objective, from sections labeled with SMI94 (myelin debris, red) and C28E10 (axon, green) (88 × 88 μm field, 2 fields per section, 2 sections per mouse). We used a custom-made two-step NIH ImageJ macro to assess the colocalization of myelin debris with axons. Myelin debris (15–20 debris per image, 4 images per mouse, 5 mice) were first randomly selected on the red channel image (blind to the green channel) and two perpendicular lines (width: 1 pixel) were drawn over each. The pixel intensity values of the red and green channels were then plotted along each of the two lines (two profiles per myelin debris). Myelin debris with a green profile peak within the red profile curve over at least one of the two lines were classified as “axon present”, whereas myelin debris not fulfilling this criterion were classified as “axon absent”. Results are expressed as a percentage of the total number of myelin debris analyzed.

#### Olig2-positive cells

The number of Olig2-positive cells was determined at the age of 20 months (*n* = 4 TgPAC-Notch3^R169C^ mice and 4 control mice). Epifluorescence images were generated from sections (1.2 ± 0.3 mm lateral to the midline) labeled with anti-Olig2 antibody, with a x20 objective, from the fimbria and the posterior part of the corpus callosum (4 sections per mouse). A custom NIH ImageJ macro was developed for the quantification of oligodendrocyte density. All steps were automated unless otherwise indicated. Briefly the global region of interest (global ROI) was delimited manually, its area was measured and the part of the image outside the global ROI was excluded. All the remaining nuclei in the DAPI channel were then binarized, separated when overlapping (watershed method) and converted into separate regions of interest (nucleus ROIs). For each nucleus ROI, mean intensity was measured for the Olig2 channel and compared with the surrounding mean intensity (mean intensity of the nucleus ROI enlarged by 70 pixels): the nucleus ROI was considered to correspond to an Olig2-positive nucleus if its mean intensity was at least 1.1 times the surrounding intensity. Oligodendrocyte density is expressed as the ratio of the total number of Olig2-positive nuclei to the area of the global ROI.

#### Microglial reaction

Total and reactive microglia were quantified in 20-month-old mice (*n* = 6 TgPAC-Notch3^R169C^ mice and 4 control mice). Epifluorescence images were generated for sections (1.5 ± 0.4 mm lateral to the midline) labeled for Iba-1 (total microglial population) and CD68 (reactive microglia) from the corpus callosum (4–6 sections per mouse), with a 40x objective. Total and reactive microglia were quantified with a custom-built NIH ImageJ macro. The corpus callosum was delimited manually, its area was measured and the part of the image outside the corpus callosum was excluded. Immunostained microglia were then binarized by automatic thresholding (the Otsu method) and the area covered by the microglia was determined. Results are expressed as the ratio of the area covered by the microglia to that of the corpus callosum.

#### Clearance of myelin debris

The phagocytosis of myelin debris by microglial cells in the corpus callosum was assessed in 20 month-old TgPAC-Notch3^R169C^ mice. Electron micrographs were acquired at a magnification of x3,400 (10–12 fields per section, 6 sections per mouse, *n* = 4 mice). Confocal images were generated for sections labeled with SMI94 and anti-Iba-1 antibodies, with a x20 oil-immersion objective (155 × 155 μm field, 4 fields per section, 2 sections per mouse, *n* = 4 mice). Myelin debris coming into contact with or internalized by microglial cell processes were counted manually on electron micrographs and on confocal images. The results are expressed as the ratio of the number of myelin debris coming into contact with or internalized by microglia to the total number of myelin debris.

### Statistical analysis

Data are expressed as the mean ± standard error of the mean (SEM). Student’s *t* tests were used for comparisons between groups. *P* values <0.05 were considered significant.

## Results

### TgPAC-Notch3^R169C^ mice display intramyelinic oedema in the brain WM

**Figure 1:**
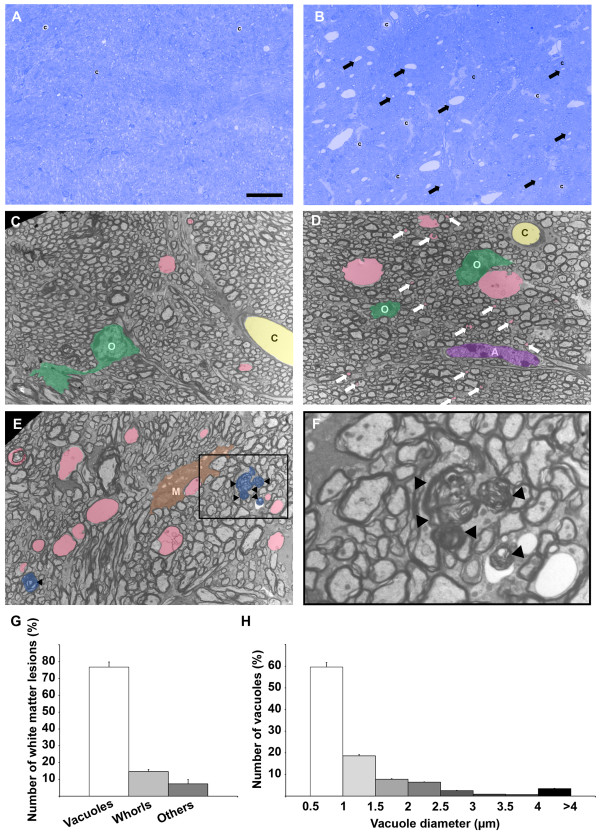
**Microvacuolation of the cerebral WM in 20-month-old TgPAC-Notch3** ^**R169C**^ **mice. (A-B)** Representative toluidine blue stained 1 μm resin sections of the corpus callosum showing abundant lesions (arrows) in the TgPAC-Notch3^R169C^ **(B)** but not in the control **(A)**. **(C-F)** Representative electron micrographs of the corpus callosum from a control **(C)** and a TgPAC-Notch3^R169C^ **(D-F)**, showing multiple membrane-bound vacuoles (**D-E**, colored in pink), including numerous inframicrometric vacuoles (**D**, white arrows), as well as myelin whorls (**E**, colored in blue and arrowheads) in the TgPAC-Notch3^R169C^ **(D, E)** contrasting with rare vacuoles in the control **(C)**. **(F)** Inset of the boxed area in **(E)** containing five myelin whorls; Inset has been left uncolored to improve visualization. **(G-H)** Diagrams showing the % of vacuoles and myelin whorls **(G)** and the distribution of vacuole diameter **(H)** in the corpus callosum of TgPAC-Notch3^R169C^ mice (n = 4), indicating that the majority of WM lesions are infra-micrometric vacuoles. O, oligodendrocytes (colored in green); A, astrocyte (colored in purple); M, microglia (colored in brown); **C**, capillary (colored in yellow). *Scale bar* represents 30 μm **(A-B)**, 5 μm **(C-E)** and 1.5 μm **(F)**.

We previously reported that TgPAC-Notch3^R169C^ mice display cerebral WM pathology upon aging [17]. We characterized this pathology further, by assessing the integrity of the cerebral WM on semithin resin sections of corpus callosum, fimbria and internal capsule from TgPAC-Notch3^R169C^ and control mice at the age of 20 months. Toluidine blue-stained semithin sections showed widespread spongiosis confined to the myelinated fiber tracts, in all regions of TgPAC-Notch3^R169C^ mice analyzed, but not in control mice (Figure 1A-B and Additional file 2: Figure S1, *n* = 4 TgPAC-Notch3^R169C^ and 4 control mice). The adjacent gray matter and the cortex of the mutant mice were almost entirely spared (Additional file 2: Figure S1).

We undertook a qualitative and quantitative electron microscopy analysis of cerebral WM in TgPAC-Notch3^R169C^ and control mice to confirm and extend these findings. We decided to use the corpus callosum for further analysis, because it contains large bundles of similarly orientated myelin fibers, making it possible to obtain cross-sections of fibers. Two predominant types of lesion were identified unambiguously: (i) membrane-bound vacuoles (Figure 1C-E, pink), which were distinguished from the vascular lumen (Figure 1C-D, yellow or “c”) by the absence of surrounding endothelial cells and astrocytic endfeet, and (ii) whorls of myelin-like figures, probably corresponding to remnants of the myelin sheath (Figure 1E-F, blue and inset). Both lesions were significantly more frequent in mutant than in control mice (Additional file 3: Figure S2, *p* < 0.05, *n* = 4 TgPAC-Notch3^R169C^ and 4 control mice). Quantitative morphological analysis showed that vacuoles were the predominant lesions in TgPAC-Notch3^R169C^ mice, accounting for 76.8 ± 5.7% of all lesions, whereas myelin whorls accounted for 14.7 ± 4.2% of all lesions (Figure 1C-G; *n* = 4 mice). Most vacuoles had a diameter of less than 1 μm (median diameter: 0.83 μm; Figure 1D, white arrows and Figure 1 H), well below the detection threshold of conventional light microscopy at about 3.5 μm, even after correction for eccentric sectioning (expected median vacuole diameter for an observed median diameter of 0.83 μm: 1.13 μm). We found no evidence of fluid accumulation in the interstitial space of TgPAC-Notch3^R169C^ mouse brains. Moreover, elements of the blood–brain barrier, including the endothelial cells with tight junctions, pericytes and astrocytic endfeet, although the latter were occasionally enlarged, were essentially morphologically normal, even when close to vacuoles (Additional file 4: Figure S3). Thus, the brain damage in aged TgPAC-Notch3^R169C^ mice consisted mostly of segmental microvacuolation of the cerebral WM tracts, and electron microscopy was required to assess this damage accurately.

**Figure 2:**
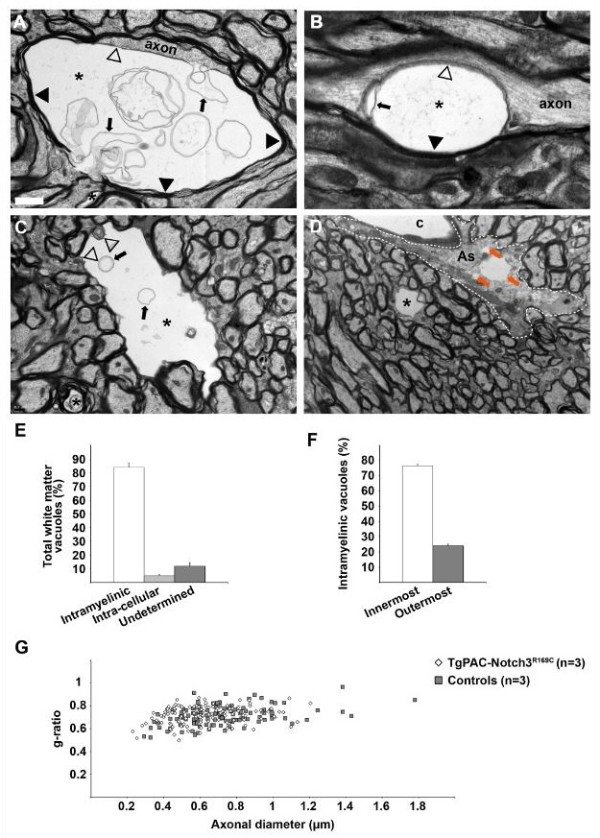
**Ultrastructural characteristics of vacuoles in the corpus callosum of TgPAC-Notch3** ^**R169C**^ **mice at 20 months of age. (A)** Typical vacuole (star) in the innermost layer of the myelin sheath. The vacuole separates the axon from its myelin sheath (black arrowheads) and contains multiple aberrant myelin sheets (arrows). Notice the thin myelin sheet (empty arrowhead) at the interface between the axon and the vacuole. **(B)** Longitudinal section of a myelinated fiber containing a vacuole in the innermost layer of the myelin sheath. **(C)** Typical vacuole (star) in the outermost layer of the myelin sheath. The vacuole is delineated by a thin rim of myelin (empty arrowheads) and contains occasional myelin debris (arrows). **(D)** Shown are 3 atypical intracellular vacuoles (orange arrows) located in an astrocytic endfoot (delineated by a white dashed line). Notice the presence of a small typical intramyelinic vacuole (star) in the same field. **(E)** Diagram of the distribution of vacuole location revealing a large preponderance of intramyelinic vacuoles (n = 4 TgPAC-Notch3^R169C^). **(F)** Diagram of the % of vacuoles developed in the innermost versus outermost layers of the myelin sheath showing a large majority of the innermost lesion type (n = 4 TgPAC-Notch3^R169C^). **(G)** Scattergram of g-ratios in relation to axonal diameters showing no difference between control (n = 4) and TgPAC-Notch3^R169C^ mice (n = 4). *Scale bar* represents 1 μm **(A)**, 0.5 μm **(B)**, 1.3 μm **(C)** and 1.75 μm **(D)**.

We looked for structural correlates of this microvacuolation by examining high-power electron micrographs of mutant corpus callosum tissue (*n* = 4 20-month-old TgPAC-Notch3^R169C^ mice). Most vacuoles (83.8 ± 3.0%) were surrounded by myelin, suggesting that they were formed within the myelin sheath (Figure 2A-C). These intra-myelinic vacuoles were generally located in the innermost layers of the myelin sheath (76.5 ± 1.1%), with the axon squashed against one side of the sheath and separated from the vacuole by a thin layer of myelin (Figure 2A, empty arrowhead). These vacuoles generally had a smooth ovoid shape, contained aberrant myelin sheets (Figure 2A, arrows) and were located in the internodal region (Figure 2A-B). They developed less frequently in the outermost layers of the myelin sheath (24 ± 1.1%), where they were surrounded by a thin layer of myelin (Figure 2C, empty arrowheads) and occasionally contained aberrant myelin sheets (Figure 2C, arrows). By contrast, 4.8 ± 1.2% of these vacuoles were located within cells, either in astrocytes (Figure 2D, orange arrows) or in microglial cells. We were unable to determine the precise subcellular location of 11.8 ± 2.6% of vacuoles, these unassigned vacuoles being extremely small (diameter ≤ 0.5 μm; Additional file 5: Figure S4).

We investigated the possibility of a general myelination problem, by determining the g-ratio for apparently intact myelin sheaths. Quantitative analysis of the g-ratio in the corpus callosum revealed no difference between TgPAC-Notch3^R169C^ and control mice (Figure 2G, *n* = 4 TgPAC-Notch3^R169C^ and 4 control mice, aged 20 months). Overall, these data indicate that segmental intramyelinic oedema, occurring preferentially in the innermost layer of the myelin sheath, can be considered to be the ultrastructural correlate of WM damage in aged TgPAC-Notch3^R169C^ mice.

### TgPAC-Notch3^R169C^ mice display progressive focal myelin alteration with poor elimination of myelin debris

**Figure 3:**
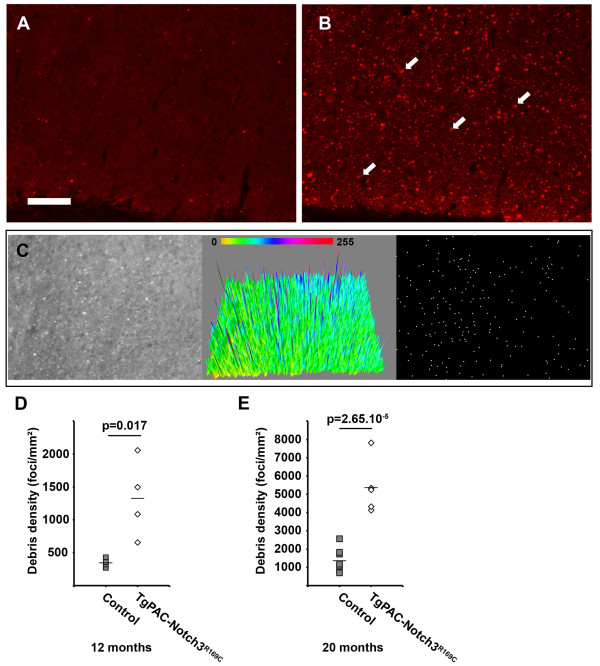
**Progressive myelin degradation in the cerebral WM of TgPAC-Notch3** ^**R169C**^ **mice. (A-B)** Representative sections of corpus callosum sections from a control **(A)** and a TgPAC-Notch3^R169C^ **(B)** immunolabeled with SMI94, which stains normal and degraded myelin; the TgPAC-Notch3^R169C^ displays numerous hyperintense foci (white arrows). **(C)** Image processing used to automatically identify the hyperintense foci. (Left panel) Shown is a 8-bit image of a TgPAC-Notch3^R169C^ corpus callosum section immunostained with SMI94. (Middle panel) Shown is the corresponding 3D image intensity profile with a LUT scale displaying the non-uniform background of the image that impedes accurate detection of hyperintense foci using simple thresholding. (Right panel) The local maxima in the image are determined to overcome this limitation: maxima are ignored if they do not stand out from the surroundings by more than a noise tolerance value (this value is set initially, from a batch of randomly selected images, to allow best discrimination of foci from background). **(D-E)** Diagrams of the myelin debris density in the corpus callosum, expressed as a total number of debris over a given area (number/mm2), in control and mutant mice at 12 months of age **(D)** (n = 4 TgPAC-Notch3^R169C^ and 4 control mice) and 20 months of age **(E)** (n = 5 TgPAC-Notch3^R169C^ and 8 control mice). Notice that the scale is different between E and D. Scale bar represents 50 μm **(A-B)**.

We evaluated the integrity of myelin further, by immunolabeling for myelin basic protein (MBP) in TgPAC-Notch3^R169C^ and control mice at 20 months of age. We used the SMI94 monoclonal antibody, which was raised against a peptide of MBP encompassing an epitope known to be unmasked during myelin degradation (Additional file 6: Figure S5) [19]. We found that this antibody, which stains normal myelin, also labeled degraded myelin with a pattern similar to but much more robust than that obtained with the polyclonal antibody commonly used to stain degraded myelin (Additional file 6: Figure S5). The labeling of brain sections with SMI94 resulted in essentially uniform staining of the WM tracts in control mice (*n* = 8; Figure 3A), whereas it revealed numerous hyperintense foci in the WM tracts, under an almost similar background of staining, in TgPAC-Notch3^R169C^ mice (*n* = 5; Figure 3B, arrows). Foci were distributed throughout all the WM tracts, including those of the corpus callosum (Figure 3B), fimbria, internal capsule and anterior commissure (data not shown), but were essentially absent from the cortex (Additional file 7: Figure S6). We quantitatively assessed myelin degradation with a quantification algorithm based on the local maxima method, which we applied to the corpus callosum of control and mutant brains labeled with SMI94 (Figure 3C). We found that there was 3.9 times more myelin debris in TgPAC-Notch3^R169C^ mice than in control mice at 20 months of age (Figure 3E, *p* = 2.6 × 10^−5^, n = 5 TgPAC-Notch3^R169C^ and 8 control mice). We assessed the progressive nature of myelin degradation in TgPAC-Notch3^R169C^ mice, by analyzing the cerebral WM of younger mice and comparing the results obtained with those for 20-month-old mice. At 12 months of age, TgPAC-Notch3^R169C^ mice had significantly larger amounts of myelin debris than aged-matched control mice (Figure 3D, *p* = 0.017, n = 4 TgPAC-Notch3^R169C^ and 4 control mice). The amount of myelin debris in TgPAC-Notch3^R169C^ mice was almost four times higher at 20 months than at 12 months, highlighting a large increase in debris load with age. Interestingly, in 12-month-old mice, myelin debris were essentially confined to the anterior periventricular region of the corpus callosum, suggesting that, as in humans, WM lesions begin in the periventricular regions, subsequently expanding towards the cortex in a centripetal manner.

It has recently been shown that the primary loss of oligodendrocytes can lead to myelinic vacuolation [20, 21]. We investigated whether the myelin damage in TgPAC-Notch3^R169C^ mice resulted from oligodendrocyte cell death, by carrying out ultrastructural examinations on mutant mice at 20 months of age. Electron micrographs showed no appreciable ultrastructural abnormalities of the oligodendrocytes in the corpus callosum (data not shown). We then evaluated oligodendrocyte density. Immunolabeling for the oligodendroglial lineage marker Olig2 showed that the density of oligodendrocytes was similar in the cerebral WM of TgPAC-Notch3^R169C^ and control mice (Additional file 8: Figure S7, *p* >0.05, *n* = 4 TgPAC-Notch3^R169C^ and 4 control mice). Furthermore, no apoptotic cells were detected on cleaved-caspase 3 immunostaining of the WM tracts of TgPAC-Notch3^R169C^ mice (data not shown).

**Figure 4:**
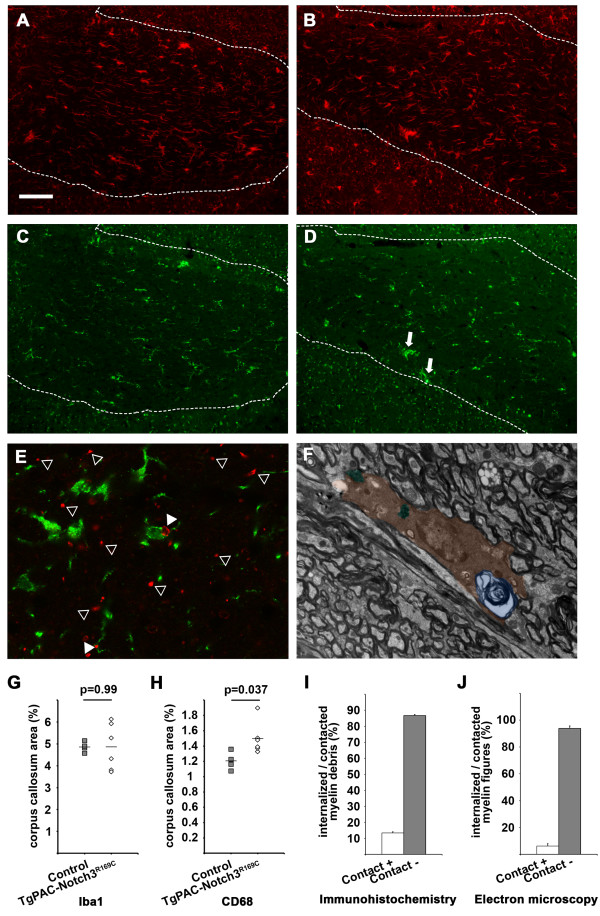
**Analysis of myelin debris clearance in the cerebral WM of TgPAC-Notch3** ^**R169C**^ **mice. (A-B)** Sections of corpus callosum (delineated by a dashed line) immunostained with anti-Iba-1 antibody showing comparable labeling patterns in the control **(A)** and TgPAC-Notch3^R169C^ **(B)** mice. **(C-D)** Sections of corpus callosum immunostained with anti-CD68 antibody showing an increased labeling and presence of microglial aggregates (arrows) in TgPAC-Notch3^R169C^ **(D)** compared to control **(C)**. **(E)** Sections of corpus callosum labeled with both SMI94 (red, myelin debris) and Iba-1 (green, microglia) showing two myelin debris contacted and/or internalized by microglial cells and processes (white arrowheads) while many others are not (empty arrowheads). **(F)** Electron micrograph of a microglial cell (colored in brown) containing a whorl of degenerating myelin (colored in blue) and two other inclusions of phagocytic material (colored in green). **(G-H)** Diagrams showing that the area of total microglia (Iba-1) is comparable between control (n = 4) and TgPAC-Notch3^R169C^ mice (n = 6) **(G)**, whereas the area of reactive microglia (CD68) is slightly, but significantly larger in TgPAC-Notch3^R169C^ (n = 6) compared to control mice (n = 4) **(H)**. **(I-J)** Diagrams showing the % of myelin debris contacted or internalized by a microglial cell as determined on confocal images **(I)** or electron micrographs **(J)** in 20-month-old TgPAC-Notch3^R169C^ mice (n = 4). *Scale bar* represents 75 μm **(A-D)**, 10 μm **(E)** and 2 μm **(F)**.

Microglia plays an essential role in removing tissue debris. There is recent evidence to suggest that the phagocytic clearance of myelin debris is required to prevent a detrimental effect of these debris on the brain and to trigger repair [22]. We began by investigating the microglial response in the cerebral WM of TgPAC-Notch3^R169C^ and control mice at 20 months of age. Immunohistochemistry with an antibody directed against Iba-1, a marker of the total microglial population, resulted in the staining of areas of similar sizes in TgPAC-Notch3^R169C^ and control mice (Figure 4A-B, G; *p* = 0.99, *n* = 6 TgPAC-Notch3^R169C^ and 4 control mice). By contrast, immunostaining for CD68, a marker of phagic microglia/macrophages, showed the CD68-positive area in TgPAC-Notch3^R169C^ mice to be slightly, but significantly larger than that in control mice (Figure 4C-D, H; *p* = 0.037, *n* = 6 TgPAC-Notch3^R169C^ and 4 control mice). We then assessed the clearance of myelin debris, by counting the number of myelin debris either in contact with or internalized by a microglial cell on brain sections labeled with SMI94 and anti-Iba-1 antibodies. In the corpus callosum, only 13.3 ± 0.7% of the myelin debris was in direct contact with microglial processes or cell bodies (Figure 4E, I; *n* = 4 TgPAC-Notch3^R169C^ mice). We confirmed this finding through another approach based on electron microscopy. An analysis of electron micrographs of the corpus callosum from TgPAC-Notch3^R169C^ mice revealed that only 6.1 ± 1.8% of abnormal myelin figures were either in contact with or internalized by a migroglial cell (Figure 4F, J, *n* = 4 TgPAC-Notch3^R169C^).

Thus, these data indicate that TgPAC-Notch3^R169C^ mice display progressive focal myelin degradation in the cerebral WM that is unlikely to result from oligodendrocyte cell death. They also suggest that this myelin pathology is associated with inefficient phagocytic clearance of the myelin debris.

### Myelin pathology in TgPAC-Notch3^R169C^ mice is associated with axon damage

**Figure 5:**
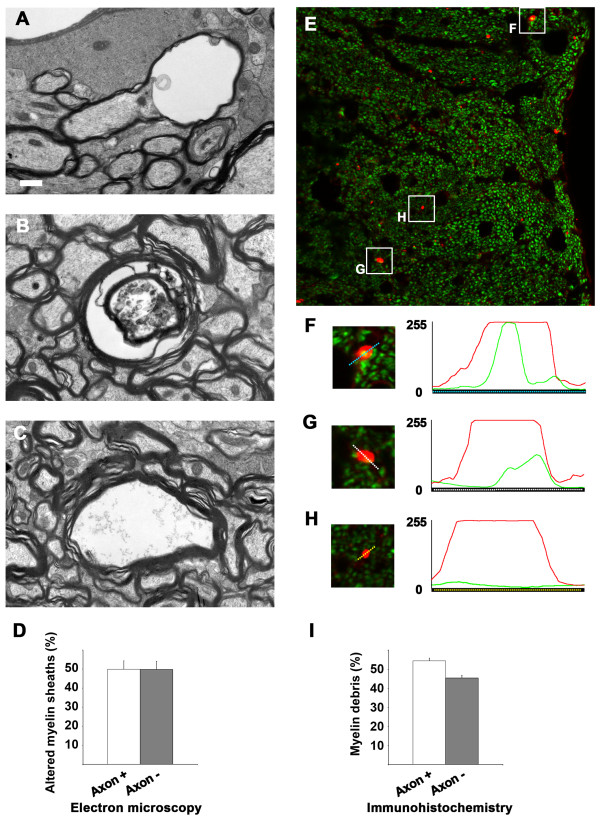
**Assessment of axonal integrity within damaged myelin sheaths in TgPAC-Notch3** ^**R169C**^ **mice aged 20 months. (A**-**C)** Representative electron micrographs of myelin sheaths containing either an apparently normal axon, although squashed against one side of the sheath **(A)** or a degenerating axon **(B)**, and of an empty sheath with no axon **(C)**. **(D)** Diagram showing that a similar proportion of abnormal myelin sheaths contains an apparently-intact axon (axon+) versus no or a degenerating axon (axon-) (n = 4 TgPAC-Notch3^R169C^ mice). **(E)** Representative confocal image of fimbria double stained with SMI94 (red, MBP hyperintense foci) and C28E10 (green, axon) antibodies. Notice that acquisition parameters have been set to minimize visualization of normal myelin and maximize visualization of axon and myelin debris. **(F-H)** Red and green profiling of MBP hyperintense foci (red) with the axon (green) in a central position **(F)** (axon+) or in lateral position **(G)** (axon +) and foci with no axon (axon-) **(H)**. **(I)** Diagram of the % of damaged myelin sheaths with axon (axon+) or without axon (axon-) (n = 5 TgPAC-Notch3^R169C^ mice). *Scale bar* represents 0.6 μm **(A, C)**, 0.5 μm **(B)** and 6.5 μm **(E)**.

We then hypothesized that axons might be disrupted either as a cause or a consequence of this prominent myelin pathology. Wallerian degeneration is a well-known process in which primary axon degeneration leads to secondary myelin sheath breakdown [23]. Conversely, in multiple sclerosis, for example, axon degeneration is thought to result from demyelination [24, 25]. We studied electron micrographs of the affected areas of WM from 20-month-old TgPAC-Notch3^R169C^ mice (*n* = 4). Three morphologically different types of lesion were observed: (i) myelin sheath vacuolation with an apparently intact axon generally flattened against one side of the sheath (Figure 5A), (ii) myelin sheath vacuolation with a degenerating axon (Figure 5B) or whorls of myelin-like figures (Figure 1E-F, blue and arrowheads) and (iii) an empty vacuolated myelin sheath with no detectable axon (Figure 5C). Quantitative analysis showed that 49.9 ± 4.4% of damaged myelin sheaths contained an apparently intact axon (lesion type (i)), whereas 49.8 ± 4.3% contained either a degenerating axon or no axon (lesions type (ii) or (iii)) (Figure 5D). We excluded from this quantitative analysis the vacuolated myelin sheaths in which vacuoles formed in the outermost lamellae of the myelin sheath, as it was not possible to determine clearly whether or not the axon was present in these sheaths. We detected no degenerating axons within morphologically normal myelin sheaths.

We also investigated the presence of axons within damaged myelin sheaths on cross-sections of fimbria labeled for phosphorylated light neurofilament and MBP in 20-month-old TgPAC-Notch3^R169C^ mice (*n* = 5; Figure 5E), to confirm these findings. Color profiling of MBP hyperintense foci on multichannel confocal images led to the identification of three distinct profiles: (i) hyperintense foci with the axon in a central position (Figure 5F), (ii) hyperintense foci with the axon in a lateral position (Figure 5G) and (iii) hyperintense foci with no axon (Figure 5H). Quantitative analysis showed that 54.5 ± 1.4% of foci were associated with an axon profile (lesion types (i) and (ii); Figure 5I). Thus, both ultrastructural and confocal analyses indicate that the myelin pathology in TgPAC-Notch3^R169C^ mice is associated with axonal damage, but that a significant proportion of damaged myelin sheaths still contain an apparently intact axon. This suggests that axonal degeneration occurs secondarily to myelin degradation.

## Discussion

This study provides, to the best of our knowledge, the first detailed microstructural characterization of the initial brain WM lesions in a well-defined form of SVD. Using a mouse model of preclinical CADASIL, we showed that myelin damage was progressive and segmental. It occurred in the absence of oligodendrocyte loss and axonal injury appeared to be secondary. Our findings identify intramyelinic oedema as a prototypic lesion. We also provide evidence for the poor clearance of myelin debris. We found no ultrastructural evidence of blood–brain-barrier leakage, providing further support for the view that prominent changes to the blood–brain barrier are not the mechanism responsible for triggering WM lesions in CADASIL [17]. Our findings shed new light on the potential mechanisms of SVD-related WM changes.

It remains unclear from the results of autopsy studies on patients with SVD whether myelin degradation, axonal injury, oligodendrocyte and neuronal loss are connected or occur sequentially [9, 26, 27]. In this study on a preclinical CADASIL model, we obtained several lines of evidence that myelin injury occurs first, in the absence of oligodendrocyte death. First, oligodendrocytes appeared normal on electron microscopy analysis, with no evidence of abnormal swelling or pyknosis. Second, Olig2 staining showed that there was no change in oligodendrocyte density in the affected WM areas. Finally, immunostaining for cleaved caspase3 revealed an absence of apoptotic oligodendrocytes. Similarly, our data suggest that axonal injury, another pathological change observed in this mouse model of CADASIL, occurs after myelin degradation. We found that about half the altered myelinated sheaths still contained an axon with no overt morphological signs of degeneration. Conversely, none of the morphologically normal myelin sheaths contained a degenerating axon. We also found that the absolute number of injured axons in affected WM areas was small. In particular, staining for non-phosphorylated neurofilaments or an axonal accumulation of APP, which is commonly used to identify and quantify injured axons [20], revealed no significant differences between mutant and control mice (data not shown). Nevertheless, additional experiments are required for the formal exclusion of more subtle axonal defects, such as changes in axonal transport, which might precede intramyelinic oedema. The lack of prominent axonal degeneration in TgPAC-Notch3^R169C^ mice may account for the lack of an overt clinical phenotype in these mice. This finding may also be of clinical relevance, because it seems likely that *NOTCH3* mutation carriers may undergo changes to the WM that remain asymptomatic until a significant number of axons have been injured or other lesions, such as lacunar infarcts, occur.

The identification of segmental intramyelinic oedema as the prototypic lesion sheds new light on the mechanisms of SVD-related WM changes, at least in CADASIL. Intramyelinic oedema is an uncommon phenotype corresponding to the accumulation of ion and water in the periaxonal space or between myelin lamellae. In myelinated axons, repetitive axonal firing leads to continuous movements of ions associated with osmotically driven movements of water. It has been suggested that the so-called “panglial syncytium”, which consists of oligodendrocytes, astrocytes and ependymal cells strongly interlinked by abundant gap junctions, is essential for the long-distance disposal of the excess ions and water released from the periaxonal space (input end) into the pericapillary and subependymal spaces (output end) [28]. This process, which is also known as spatial potassium buffering or siphoning, presumably prevents axonal activation-induced intramyelinic oedema. There is strong evidence, from both mouse models and human diseases, that changes to the molecular components of this panglial syncytium, from the input to the output ends, can ultimately cause ultrastructural lesions of the myelin, with intramyelinic oedema. Mice in which the gap junction coupling of oligodendrocytes and astrocytes is eliminated by the genetic inactivation of both connexins on oligodendrocytes (Cx32 and Cx47) or astrocytes (Cx30 and Cx43) display myelin-associated vacuoles in the cerebral WM tracts, with intramyelinic oedema. This phenotype is greatly attenuated by the suppression of axonal activity, and strongly exacerbated by increases in neuronal activity [29, 30]. In mice, deletion of the gene encoding the potassium channel Kir4.1 [31] or the gene encoding the chloride channel ClC-2 [32], both of which are strongly expressed in the astrocytic endfeet at the perivascular basal lamina, the output end of the panglial syncytium, leads to WM vacuolation with intramyelinic oedema. Kir4.1 is a weakly rectifying potassium channel, capable of mediating spatial potassium buffering in the central nervous system [33]. There is genetic evidence in mice to suggest that Kir4.1 and connexins are involved in the same pathway [29, 30]. Finally, in humans, a loss of ClC-2 function and dominant mutations of the gene encoding MLC1, a protein that is also highly abundant in the astrocytic endfeet, cause megalencephalic leukoencephalopathy with subcortical cysts, a disease characterized by chronic brain WM oedema in vacuoles predominantly located within myelin sheaths [34, 35]. These findings, together with those presented here, support the notion that early WM lesions in CADASIL may be due to a defect in ion and water homeostasis, providing a new starting point for mechanistic studies.

Mutant NOTCH3 could impair water and ion homeostasis via at least two potential mechanisms. Firstly, chronic hypoperfusion, which has been demonstrated in TgPAC-Notch3^R169C^ mice [17], may decrease the functioning of ion pumps involved in ionic homeostasis, leading to intramyelinic oedema. Early studies in rodents showed that the cerebral WM was highly vulnerable to ischemia [36, 37]. Remarkably, segmental vacuolation of the WM tracts, with intramyelinic oedema, is one of the most conspicuous and very early pathological changes in ischemic WM induced by brief focal ischemia, in addition to major damage to oligodendrocytes and axons [37]. An energy deficit leads to a decrease in ATP levels, resulting in the failure of several key ATP-dependent ion pumps thought to make a critical contribution to overall ion homeostasis [38]. Alternatively, the mutant NOTCH3 may impair the functioning of astrocytic endfeet, the output end of the panglial syncytium, directly. The astrocytic endfeet cover the abluminal surface of pericytes and smooth muscle cells where mutant NOTCH3 accumulates aberrantly [17, 39]. Further experimental studies are required to test these hypotheses.

We found that myelin degradation was associated with a weak microglial response. Moreover, negligible amounts of myelin debris were eliminated by phagocytosis, as demonstrated by both immunohistochemical and electron microscopy studies, suggesting that the resident microglia was inefficient at the removal of myelin debris. The reasons for this poor clearance of debris remain unclear. Inefficient microglial clearance has been reported to be prevalent in several neurodegenerative diseases and to increase with aging [22]. It has also been suggested that resident microglia clear debris less efficiently in the presence of an intact blood–brain barrier, because of the absence of blood-derived macrophages or stimulation by recruited adaptive immune cells [22]. The clearance of tissue debris is thought to play an important role in creating a proregenerative environment within the central nervous system. The presence of uncleared myelin debris in the central nervous system has been associated with inefficient remyelination and impaired axon regeneration [22, 23].

Our study has several methodological strengths. These include the use of a genetically defined model of SVD in which no surgery was required to obtain the WM phenotype. Bilateral carotid artery stenosis or ligation is commonly used to reproduce mild chronic hypoperfusion and associated ischemic injury to the WM. This model has the disadvantage of requiring a surgical procedure that may itself cause various degrees of transient brain ischemia potentially leading to brain lesions [36, 40]. We assessed the focal degradation of myelin by a quantitative and semi-automatic approach that we developed, which proved to be highly sensitive and robust, with very low interobserver variability (data not shown). This approach has clear advantages over other widely used qualitative or semi-quantitative approaches [19, 41]. Finally, electron microscopy was found to be crucial for the evaluation of vacuolation.

However, this study also has several limitations. We cannot exclude the possibility that WM lesions in CADASIL differ markedly from those in other forms of SVD. However, many studies have highlighted the similarities of WM lesions between patients with CADASIL and those with age- or hypertension-related SVD, particularly as concerns the overall spread of changes across disease stages [42]. We did not attempt to assess cognitive functions in the TgPAC-Notch3^R169C^ mice. A recurrent issue concerning WM changes is their possible association with cognitive deficits, including executive dysfunction in particular, and disability, and the strength of the correlation. However, TgPAC-Notch3^R169C^ mice were generated in the genetic FVB/N background. This background is widely used for genetic manipulations, but behavioral assessments in mice of this strain are complicated due to their relative hyperactivity and severe visual impairment [43]. Finally, it may be of interest to apply novel MRI techniques to TgPAC-Notch3^R169C^ mice, to define neuroradiological correlates of these early WM lesions in CADASIL.

## Conclusion

Using TgPAC-Notch3^R169C^ mice as a model of a pure, well defined SVD, we identified intramyelinic oedema as an early and conspicuous feature of cerebral WM changes. Our findings suggest that SVD-related WM changes may be triggered by a defect in ion and water homeostasis. Our results thus provide a new starting point for mechanistic studies of the pathogenesis of SVD–related WM changes.

### Supporting data

The data sets supporting the results of this article are included within the article and its additional files.

## Electronic supplementary material

Additional file 1: Table S1: Antibodies used in specific applications. (DOCX 13 KB)

Additional file 2: Figure S1: Assessment of cerebral lesions in TgPAC-Notch3^R169C^ mice by light microscopy on semi-thin resin sections. Representative toluidine-blue stained 1 μm semi-thin resin sections of fimbria (A, B), internal capsule (C, D) and cortex (E, F) from control (A, C, E) and TgPAC-Notch3^R169C^ (B,D,F) mice. White matter (WM) tracts of fimbria and internal capsule exhibit widespread spongiosis (arrows) in TgPAC-Notch3^R169C^ (B, D) comparatively to control (A, C), whereas the cortex of TgPAC-Notch3^R169C^ is spared (F). c, capillary lumen. Scale bar represents 20 μm. Representative out of 4 TgPAC-Notch3^R169C^ and 4 control mice aged 20 months. (TIFF 19 MB)

Additional file 3: Figure S2: Quantitative assessment of electron microscopy lesion load in the cerebral WM. Diagrams showing the number of WM lesions (A) and vacuoles (B) in the corpus callosum of 20-month-old control (n = 4) and TgPAC-Notch3^R169C^ mice (n = 4) as determined on electron micrographs. Amount of WM lesions and vacuoles is significantly increased in TgPAC-Notch3^R169C^ compared to control. (TIFF 8 MB)

Additional file 4: Figure S3: Electron microscopy assessment of the blood brain barrier in the WM from TgPAC-Notch3^R169C^ mice. Shown are representative electron micrographs of WM capillaries from TgPAC-Notch3^R169C^ mice, at medium (A) and high magnification (B) showing no overt abnormality of the elements of the blood brain barrier including tight junctions of endothelial cells (white arrowheads), basement membrane, pericytes (P) and astrocytic endfeet (As). Notice the presence of a typical intramyelinic vacuole (star) in the vicinity of the capillary (A). Scale bar represents 1 μm (A) and 0.15 μm (B). Representative out of 4 TgPAC-Notch3^R169C^ mice aged 20 months. (TIFF 8 MB)

Additional file 5: Figure S4: WM vacuoles of undetermined location. Shown are two representative electron micrographs of WM from TgPAC-Notch3^R169C^ mice with both typical intramyelinic vacuoles (star) and vacuoles of uncertain subcellular location (arrows). These “unassigned” vacuoles are usually very small (mean diameter, 0.25 μm), membrane bound (A) and tend to coalesce (B). Scale bar represents 0.5 μm. Representative out of 4 TgPAC-Notch3^R169C^ mice aged 20 months. (TIFF 2 MB)

Additional file 6: Figure S5: SMI94 labels degraded myelin. (A) Sequence alignment of guinea pig and human myelin basic protein (MBP) showing that SMI94 monoclonal antibody and the historical anti-degraded MBP polyclonal antibody (dMBP) have been raised against overlapping epitopes. (B-C) Shown are adjacent sections of the corpus callosum from 20-month-old TgPAC-Notch3^R169C^ mice immunostained with anti-dMBP (B) or SMI94 (C) displaying comparable staining pattern of hyperintense foci. (D-L) Shown are adjacent corpus callosum sections from a 20-month-old TgPAC-Notch3^R169C^ mouse double labeled with anti-dMBP (green, left panel) and SMI-94 (red, middle panel) at the indicated dilutions and the corresponding merged picture (right panel). (D-F) anti-dMBP and SMI94 antibodies were used at the same dilution than in B and C. Notice that almost all green and red hyperintense foci co-localize (F), and that the staining intensity with anti-dMBP (D) is strongly reduced when this antibody is used in combination with SMI94. (G-I) A 5-fold decrease in SMI94 concentration partially restores anti-dMBP staining intensity while (J-L) a 5-fold decrease in anti-dMBP concentration almost abolishes anti-dMBP staining. Scale bar represents 50 μm. Representative out of 3 TgPAC-Notch3^R169C^ mice aged 20 months. (TIFF 19 MB)

Additional file 7: Figure S6: SMI94 specifically marks degraded myelin in TgPAC-Notch3^R169C^ mice. Photomicrographs of SMI94 immunostaining from the head of the corpus callosum (cc) and adjacent cortex of representative controls (A) and TgPAC-Notch3^R169C^ mice (B). WM tracts in the control (A) are uniformly stained, whereas WM tracts in TgPAC-Notch3^R169C^ (B) display numerous hyperintense foci. Scale bar represents 100 μm. White dashed line delineate the corpus callosum from the cortex. Representative out of 3 control and 3 TgPAC-Notch3^R169C^ mice aged 20 months. (TIFF 19 MB)

Additional file 8: Figure S7: Quantitative assessment of oligodendrocytes in TgPAC-Notch3^R169C^ mice WM. (A-B) Representative fimbria sections from control (A) and TgPAC-Notch3^R169C^ (B) mice immunostained with anti-Olig2 (green) antibody, with nucleus counterstained with DAPI (blue). (C-D) Diagrams of oligodendrocyte density in the fimbria (C) and in the posterior part of the corpus callosum (D) showing comparable oligodendrocyte density in control (n = 4) and TgPAC-Notch3^R169C^ mice (n =4) at 20 months of age. Scale bar represents 100 μm. (TIFF 19 MB)

## Abbreviations

BSA: Bovine serum albumin
CADASIL: Cerebral Autosomal Dominant Arteriopathy with Subcortical Infarcts and Leukoencephalopathy
DTI: Diffusion Tensor Imaging
MRI: Magnetic resonance imaging
MTI: Magnetization transfer imaging
PB: Sodium phosphate buffer
SVD: Small Vessel Disease
WM: White matter.
