# Supplementary material for: Early white matter changes in CADASIL: evidence of segmental intramyelinic oedema in a pre-clinical mouse model
Source: Acta Neuropathol Commun. 2014 Apr 30;2:49. doi: 10.1186/2051-5960-2-49 (PMC4035092; doi:10.1186/2051-5960-2-49)
Supplement: Supplementary file 1 — Additional file 1: Table S1: Antibodies used in specific applications. (DOCX 13 KB) [file 40478_2014_123_MOESM1_ESM.docx]

**Antibodies used in specific applications**

| **Primary Antibody**  **(dilution used, clone, Manufacture)** | **Tissue processing** | **Secondary antibody**  **(Manufacture, catalog #, dilution used)** |
| --- | --- | --- |
| **Myelin markers** |  |  |
| mouse monoclonal anti-myelin Basic Protein (1:2000, SMI94, Covance) | Free floating cryosections (16 µm) | Alexa fluor 594-conjugated goat anti-mouse, highly cross-adsorbed  (Life technologies, 1:500) |
| rabbit polyclonal anti-degraded myelin basic protein (1:500, 1:2500, Millipore), | Free floating cryosections (16 µm) | Alexa fluor 488-conjugated goat anti-rabbit  (Life technologies, 1:500) |
| **Microglia markers** |  |  |
| rabbit polyclonal anti-Iba1 (1:2000, Wako), | Free floating cryosections (16 µm) | Alexa fluor 594-conjugated goat anti-rabbit  (Life technologies, 1:500) |
| rat monoclonal anti-CD68 (1:250, FA-11, AbD Serotec) | Free floating cryosections (16 µm) | Alexa fluor 488-conjugated goat anti-rat  (Life technologies, 1:500) |
| **Axonal markers** |  |  |
| rabbit monoclonal anti-phosphorylated neurofilament light (1:100, C28E10, Cell Signaling). | Free floating cryosections (16 µm) | Alexa fluor 488-conjugated goat anti-rabbit  (Life technologies, 1:500) |
| mouse monoclonal anti-non-phosphorylated neurofilament heavy (1:50000, SMI32, Covance) | Free floating cryosections (16 µm) | Alexa fluor 594-conjugated goat anti-mouse, highly cross-adsorbed  (Life technologies, 1:500) |
| mouse monoclonal anti-Alzheimer precursor protein A4 (1:1000, 22C11, Millipore) | Free floating cryosections (16 µm) | Alexa fluor 594-conjugated goat anti-mouse, highly cross-adsorbed  (Life technologies, 1:500) |
| **Oligodendrocyte marker** |  |  |
| rabbit polyclonal anti-Olig2 antibody (1:200, Millipore) | paraffin sections (7 µm), citrate buffer unmasking | Alexa fluor 488-conjugated goat anti-rabbit  (Life technologies, 1:500) |
| **Apoptosis marker** |  |  |
| rabbit monoclonal anti-cleaved-caspase 3 (1:250, Asp175 / 5A1E, Cell Signaling) | Free floating cryosections (16 µm) | Alexa fluor 488-conjugated goat anti-rabbit  (Life technologies, 1:500) |
