# Supplementary figures and images for: Early white matter changes in CADASIL: evidence of segmental intramyelinic oedema in a pre-clinical mouse model
Source: Acta Neuropathol Commun. 2014 Apr 30;2:49. doi: 10.1186/2051-5960-2-49 (PMC4035092; doi:10.1186/2051-5960-2-49)

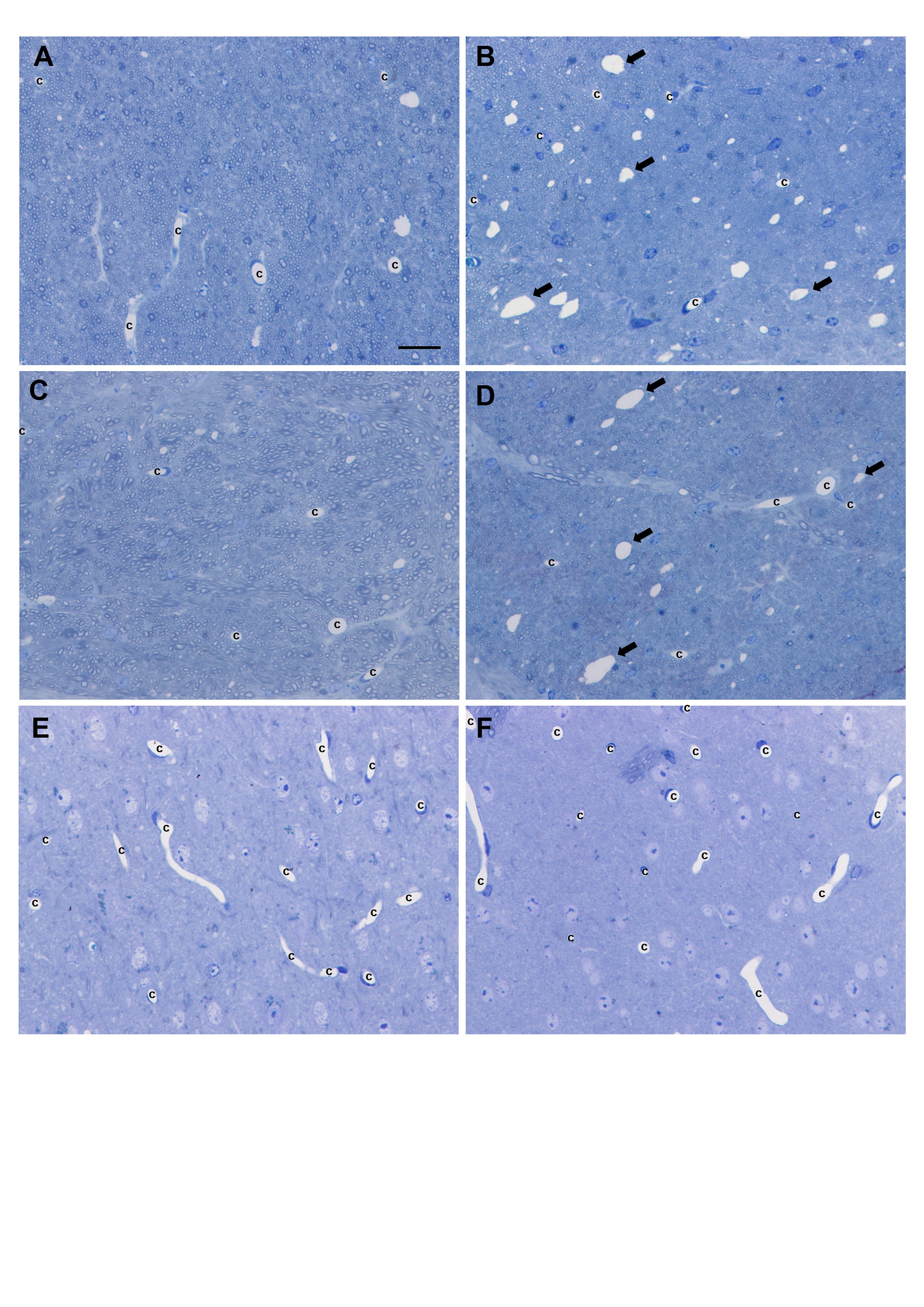

Supplement: Supplementary file 2 — Additional file 2: Figure S1: Assessment of cerebral lesions in TgPAC-Notch3R169C mice by light microscopy on semi-thin resin sections. Representative toluidine-blue stained 1 μm semi-thin resin sections of fimbria (A, B), internal capsule (C, D) and cortex (E, F) from control (A, C, E) and TgPAC-Notch3R169C (B,D,F) mice. White matter (WM) tracts of fimbria and internal capsule exhibit widespread spongiosis (arrows) in TgPAC-Notch3R169C (B, D) comparatively to control (A, C), whereas the cortex of TgPAC-Notch3R169C is spared (F). c, capillary lumen. Scale bar represents 20 μm. Representative out of 4 TgPAC-Notch3R169C and 4 control mice aged 20 months. (TIFF 19 MB) [file 40478_2014_123_MOESM2_ESM.tiff]

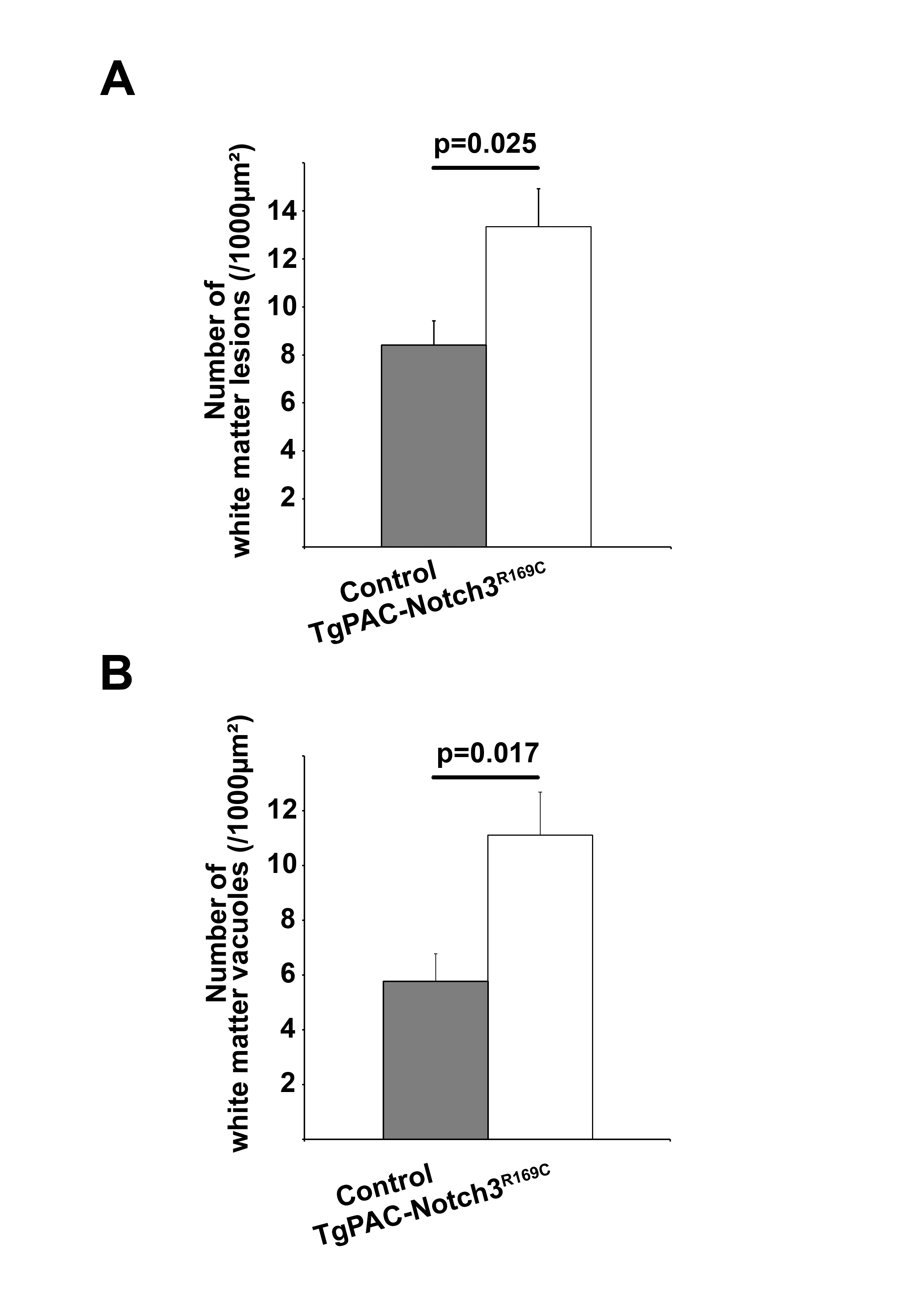

Supplement: Supplementary file 3 — Additional file 3: Figure S2: Quantitative assessment of electron microscopy lesion load in the cerebral WM. Diagrams showing the number of WM lesions (A) and vacuoles (B) in the corpus callosum of 20-month-old control (n = 4) and TgPAC-Notch3R169C mice (n = 4) as determined on electron micrographs. Amount of WM lesions and vacuoles is significantly increased in TgPAC-Notch3R169C compared to control. (TIFF 8 MB) [file 40478_2014_123_MOESM3_ESM.tiff]

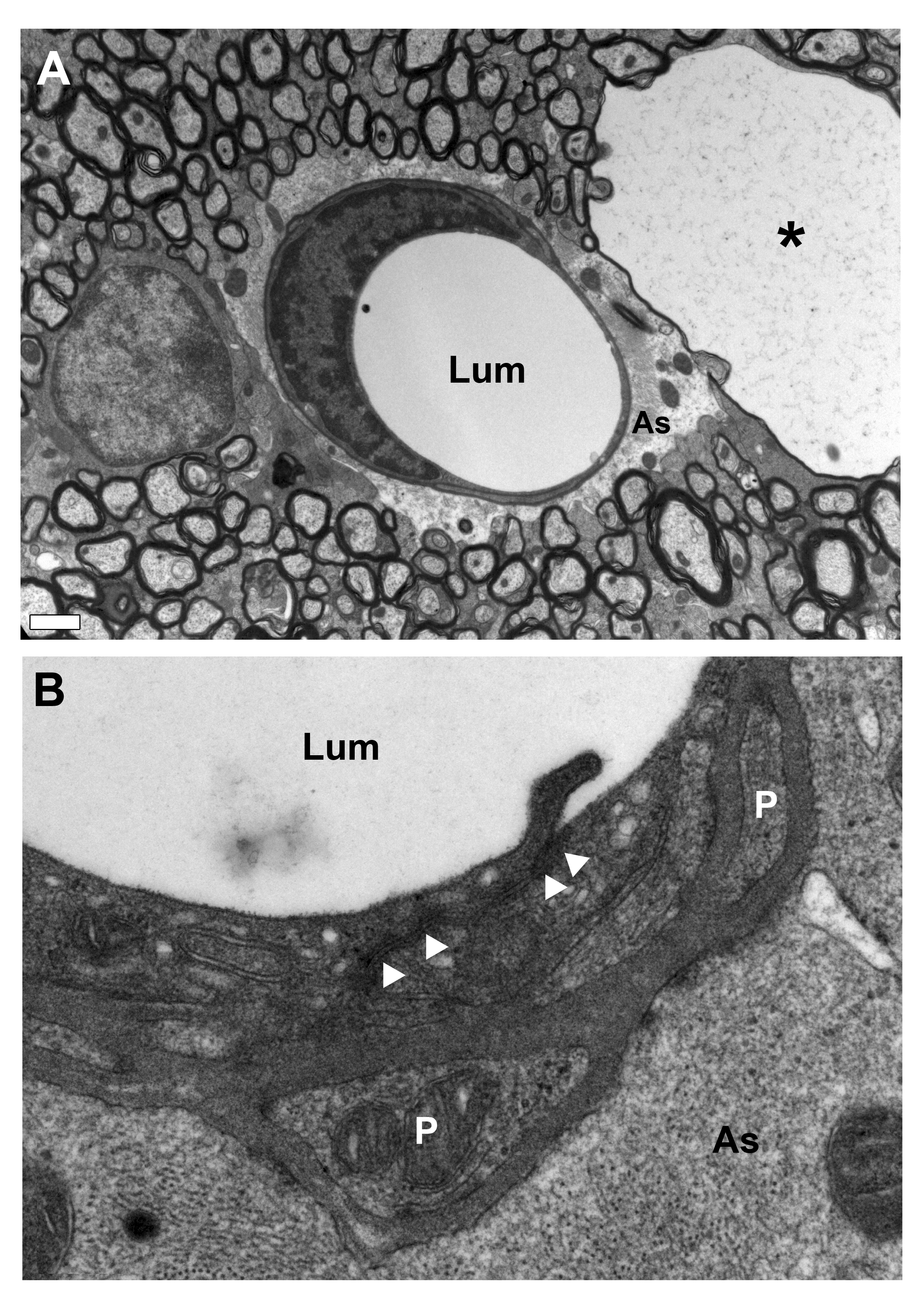

Supplement: Supplementary file 4 — Additional file 4: Figure S3: Electron microscopy assessment of the blood brain barrier in the WM from TgPAC-Notch3R169C mice. Shown are representative electron micrographs of WM capillaries from TgPAC-Notch3R169C mice, at medium (A) and high magnification (B) showing no overt abnormality of the elements of the blood brain barrier including tight junctions of endothelial cells (white arrowheads), basement membrane, pericytes (P) and astrocytic endfeet (As). Notice the presence of a typical intramyelinic vacuole (star) in the vicinity of the capillary (A). Scale bar represents 1 μm (A) and 0.15 μm (B). Representative out of 4 TgPAC-Notch3R169C mice aged 20 months. (TIFF 8 MB) [file 40478_2014_123_MOESM4_ESM.tiff]

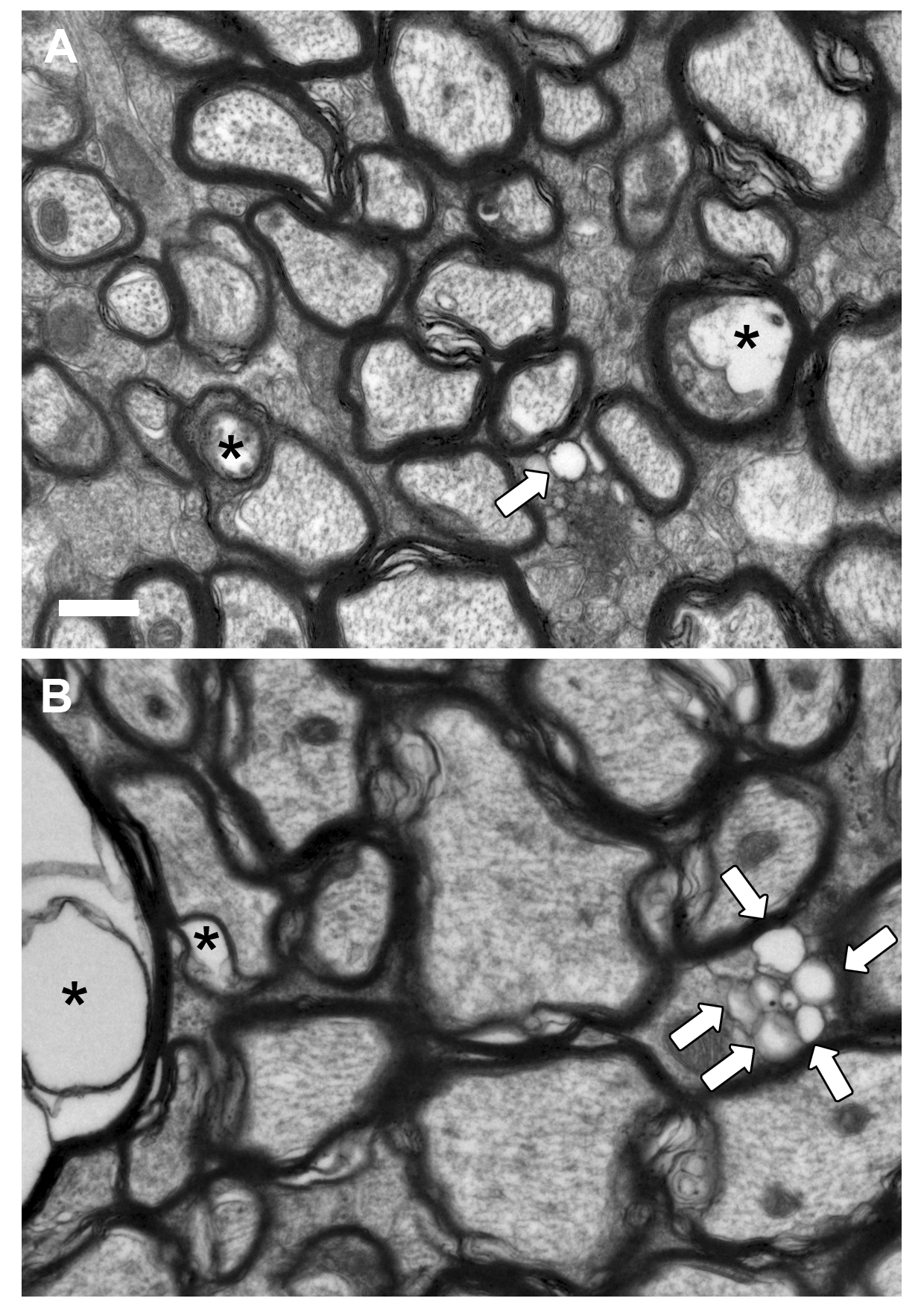

Supplement: Supplementary file 5 — Additional file 5: Figure S4: WM vacuoles of undetermined location. Shown are two representative electron micrographs of WM from TgPAC-Notch3R169C mice with both typical intramyelinic vacuoles (star) and vacuoles of uncertain subcellular location (arrows). These “unassigned” vacuoles are usually very small (mean diameter, 0.25 μm), membrane bound (A) and tend to coalesce (B). Scale bar represents 0.5 μm. Representative out of 4 TgPAC-Notch3R169C mice aged 20 months. (TIFF 2 MB) [file 40478_2014_123_MOESM5_ESM.tiff]

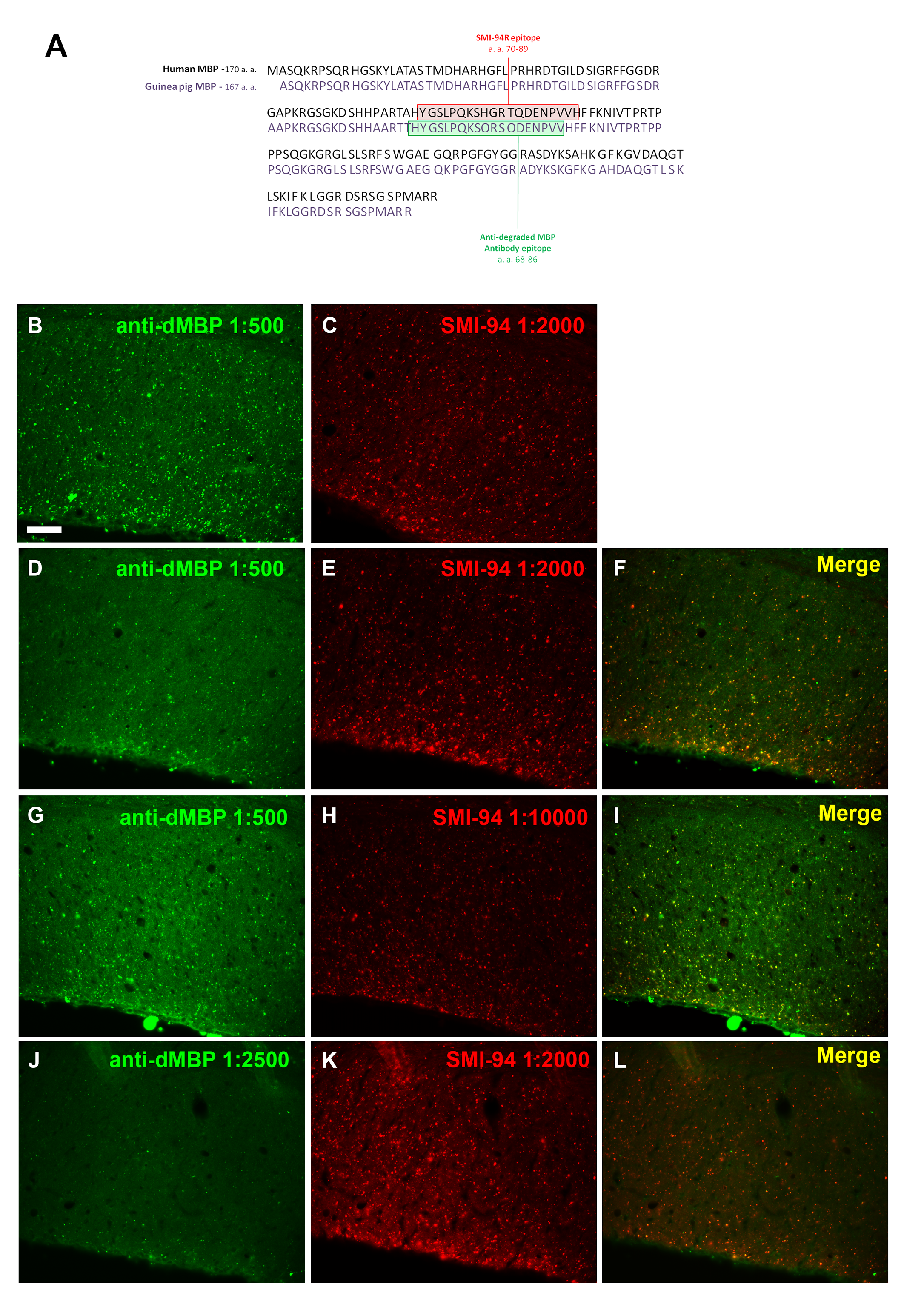

Supplement: Supplementary file 6 — Additional file 6: Figure S5: SMI94 labels degraded myelin. (A) Sequence alignment of guinea pig and human myelin basic protein (MBP) showing that SMI94 monoclonal antibody and the historical anti-degraded MBP polyclonal antibody (dMBP) have been raised against overlapping epitopes. (B-C) Shown are adjacent sections of the corpus callosum from 20-month-old TgPAC-Notch3R169C mice immunostained with anti-dMBP (B) or SMI94 (C) displaying comparable staining pattern of hyperintense foci. (D-L) Shown are adjacent corpus callosum sections from a 20-month-old TgPAC-Notch3R169C mouse double labeled with anti-dMBP (green, left panel) and SMI-94 (red, middle panel) at the indicated dilutions and the corresponding merged picture (right panel). (D-F) anti-dMBP and SMI94 antibodies were used at the same dilution than in B and C. Notice that almost all green and red hyperintense foci co-localize (F), and that the staining intensity with anti-dMBP (D) is strongly reduced when this antibody is used in combination with SMI94. (G-I) A 5-fold decrease in SMI94 concentration partially restores anti-dMBP staining intensity while (J-L) a 5-fold decrease in anti-dMBP concentration almost abolishes anti-dMBP staining. Scale bar represents 50 μm. Representative out of 3 TgPAC-Notch3R169C mice aged 20 months. (TIFF 19 MB) [file 40478_2014_123_MOESM6_ESM.tiff]

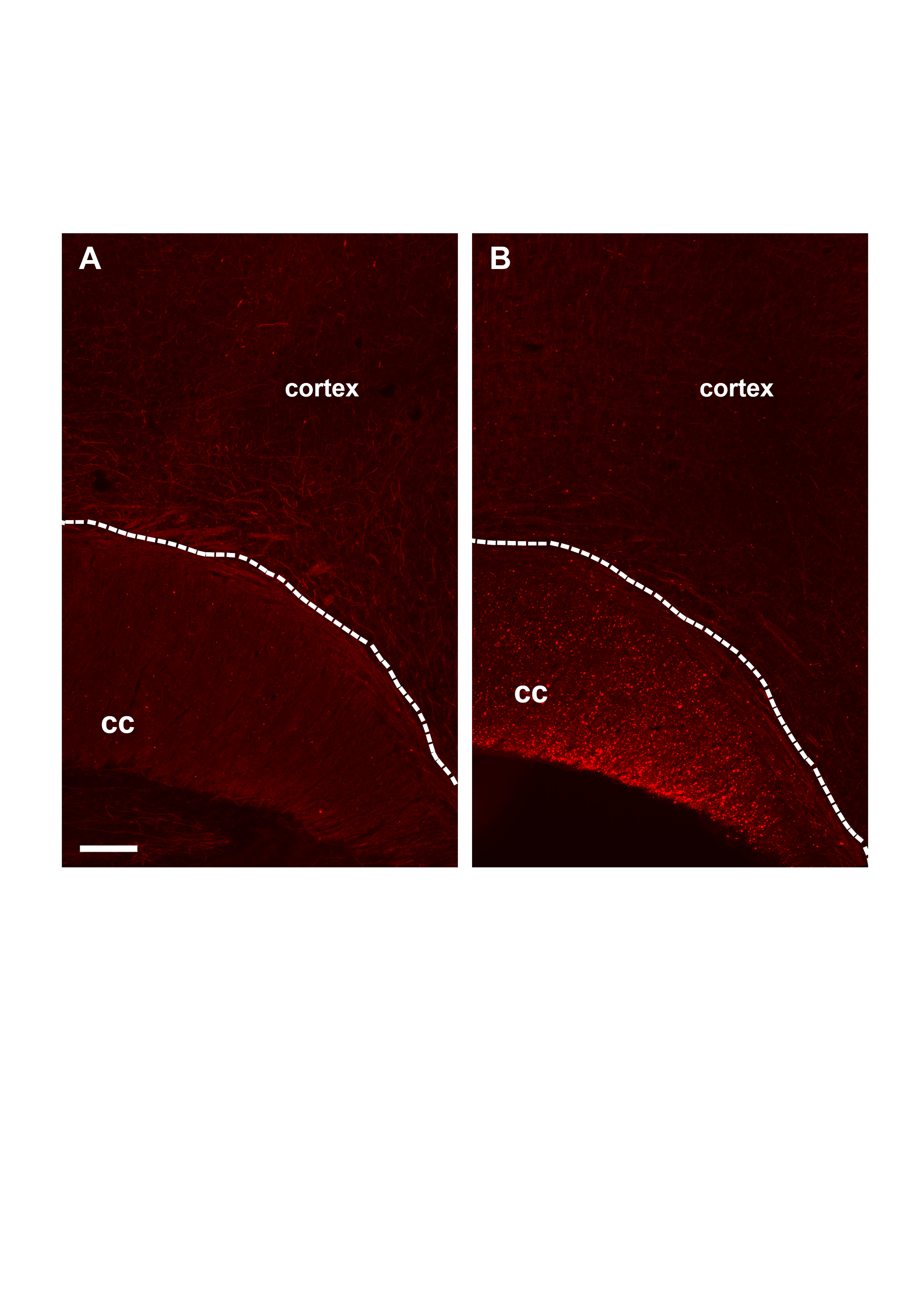

Supplement: Supplementary file 7 — Additional file 7: Figure S6: SMI94 specifically marks degraded myelin in TgPAC-Notch3R169C mice. Photomicrographs of SMI94 immunostaining from the head of the corpus callosum (cc) and adjacent cortex of representative controls (A) and TgPAC-Notch3R169C mice (B). WM tracts in the control (A) are uniformly stained, whereas WM tracts in TgPAC-Notch3R169C (B) display numerous hyperintense foci. Scale bar represents 100 μm. White dashed line delineate the corpus callosum from the cortex. Representative out of 3 control and 3 TgPAC-Notch3R169C mice aged 20 months. (TIFF 19 MB) [file 40478_2014_123_MOESM7_ESM.tiff]

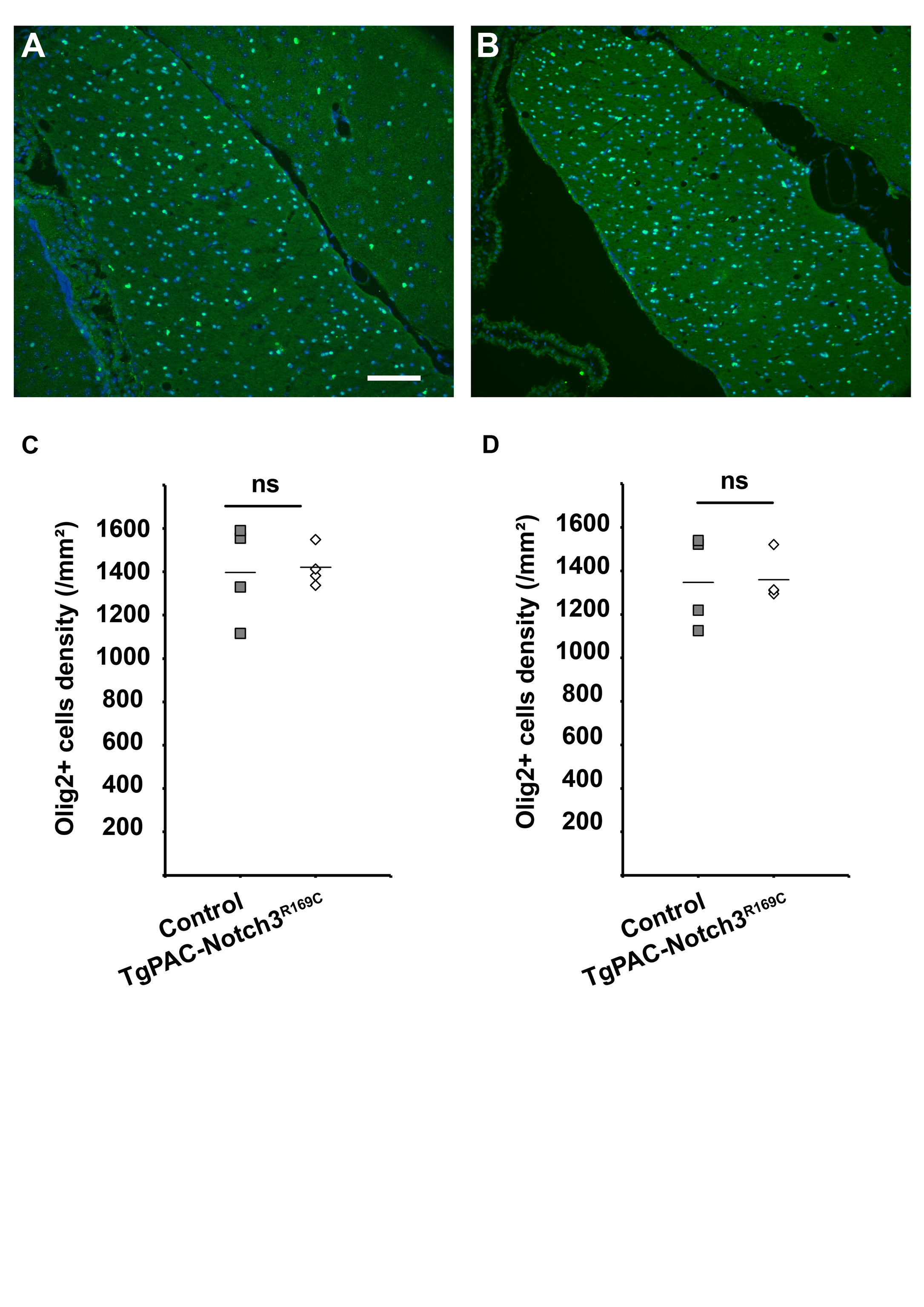

Supplement: Supplementary file 8 — Additional file 8: Figure S7: Quantitative assessment of oligodendrocytes in TgPAC-Notch3R169C mice WM. (A-B) Representative fimbria sections from control (A) and TgPAC-Notch3R169C (B) mice immunostained with anti-Olig2 (green) antibody, with nucleus counterstained with DAPI (blue). (C-D) Diagrams of oligodendrocyte density in the fimbria (C) and in the posterior part of the corpus callosum (D) showing comparable oligodendrocyte density in control (n = 4) and TgPAC-Notch3R169C mice (n =4) at 20 months of age. Scale bar represents 100 μm. (TIFF 19 MB) [file 40478_2014_123_MOESM8_ESM.tiff]
